# Solid‐State Transformation of Amorphous Calcium Carbonate to Aragonite Captured by CryoTEM

**DOI:** 10.1002/anie.201703158

**Published:** 2017-08-15

**Authors:** Jessica M. Walker, Bartosz Marzec, Fabio Nudelman

**Affiliations:** ^1^ School of Chemistry University of Edinburgh Joseph Black Building David Brewster Road Edinburgh EH9 3FJ UK

**Keywords:** biomineralization, calcium carbonate, cryo-electron microscopy, crystal growth, non-classical crystallization

## Abstract

Early‐stage reaction mechanisms for aragonite‐promoting systems are relatively unknown compared to the more thermodynamically stable calcium carbonate polymorph, calcite. Using cryoTEM and SEM, the early reaction stages taking place during aragonite formation were identified in a highly supersaturated solution using an alcohol–water solvent, and an overall particle attachment growth mechanism was described for the system. In vitro evidence is provided for the solid‐state transformation of amorphous calcium carbonate to aragonite, demonstrating the co‐existence of both amorphous and crystalline material within the same aragonite needle. This supports non‐classical formation of aragonite within both a synthetic and biological context.

Calcium carbonate is a frequently used model mineral in studies of crystallization mechanisms,^1^ due to its availability in nature, wide‐spread application as a construction material for hard tissues, and pivotal role in the global carbon cycle. Calcite, which is the thermodynamically stable polymorph of calcium carbonate, is the usual product of CaCO_3_ crystallization performed at low supersaturations. Precipitation of the kinetically privileged polymorphs (amorphous calcium carbonate (ACC), ikaite, vaterite, or aragonite) is achievable either by increasing the supersaturation of the growth mixture, changing the crystallization temperature, or by introducing additives capable of selecting a particular polymorph.^2^ An exemplary system of such an additive frequently used in synthetic crystallization experiments is Mg^2+^ ions, whose presence in the growth solution facilitates the formation of aragonite. ACC and vaterite can be precursors to calcite, as well as being reaction products themselves when stabilized by kinetic control.^3^

Two main models of formation to describe the different routes by which CaCO_3_ forms have been proposed: classical and non‐classical. Classical growth is through ion‐by‐ion attachment, beginning from a nucleus of a critical size. Non‐classical growth describes the aggregation of more complex particles, ranging from multi‐ion complexes to nanocrystals.^4^ This assembly has been shown to occur through both oriented and non‐oriented attachment as well as amorphous addition.^5^ Recent evidence suggests more than one crystallization pathway can operate in a single system, including both classical and non‐classical features.^6^

Although mechanisms of calcite growth are well studied, aragonite pathways are less well‐known. This polymorph has been shown to form via an amorphous precursor both in synthetic and biological systems,^7^ and while a non‐classical crystal growth process has been suggested, the mechanisms of aragonite formation are still unknown. Here, we used a water and alcohol mixture to stabilize the formation of aragonite to study the crystallization mechanisms using cryo‐transmission electron microscopy (cryoTEM). This technique allows the direct visualization of the early stages of the crystallization process while maintaining intermediate reaction products in their native, hydrated state.^8^ Utilizing this method, we demonstrate that aragonite formation in this highly supersaturated system progresses via amorphous precursor particles that aggregate to form a crystal, going through an intermediate in which semi‐oriented crystalline domains co‐exist with the amorphous phase. This provides evidence for an amorphous‐to‐crystalline solid state transformation that, while known for other calcium carbonate polymorphs,^9^ has not been previously observed for aragonite.

To form aragonite, CaCl_2_⋅2 H_2_O was added to Na_2_CO_3_ to form a final reaction concentration of 0.025 m with ethanol as 50 vol % of the solvent, at a gentle shaking speed.^10^ To prepare samples for cryoTEM, 3 μL of reaction solution was immediately vitrified at the required timepoint. For SEM, 10 μL of solution was filtered through a 0.22 μm membrane which was carbon coated for imaging (Methods, Supporting Information).

Two pre‐crystalline stages could be identified by cryoTEM. Firstly, after three minutes of reaction, particles of 60–100 nm in size were present (Figure 1 a, arrows). The corresponding diffraction pattern confirms that these were ACC (Figure 1 a, inset). Interestingly, most of the ACC particles formed a network in the solution, rather than being individually dispersed. After 10 minutes, larger particles of 250–300 nm in size were present and remained amorphous (Figure 1 b and LDSAED, inset). These large ACC particles were further aggregated into clusters of particles (Figure 1 b, arrow) and aligned into elongated structures, suggesting that particle attachment was taking place. These stages can also be observed in the corresponding SEM images. Figure 2 a shows small spherical ACC particles of 75–100 nm after one minute (higher magnification shown inset), corresponding to the first stage observed by cryoTEM (Figure 1 a).

**Figure 1:**
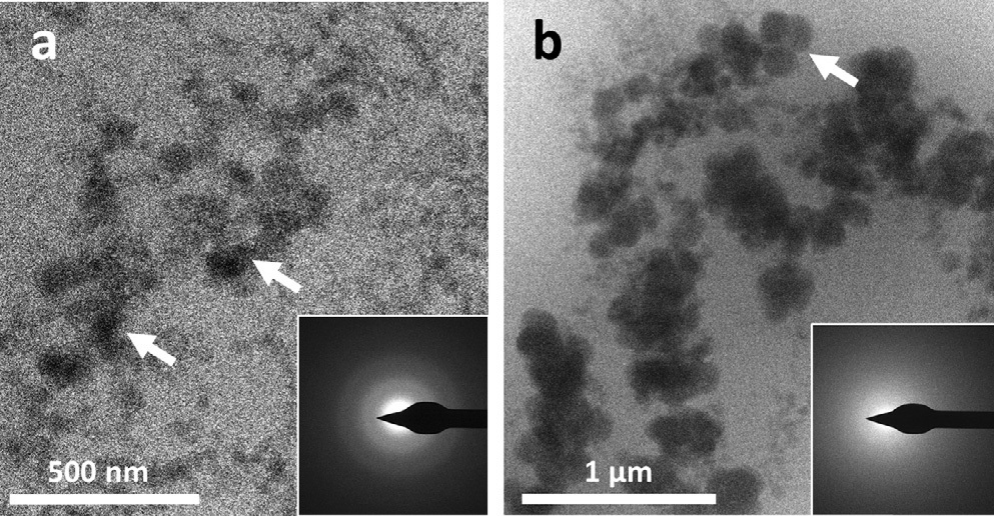
CryoTEM images of reaction solution: a) after 3 minutes of reaction. A network of small (60–120 nm) ACC is visible. Inset: Low‐dose selected‐area electron‐diffraction (LDSAED) showing that the particles are composed of ACC and b) after 10 minutes of reaction. Larger ACC (250–300 nm) is forming and assembling into linear structures. Arrow: A cluster of particles that has formed. Inset: LDSAED showing that the particles are composed of ACC.

**Figure 2:**
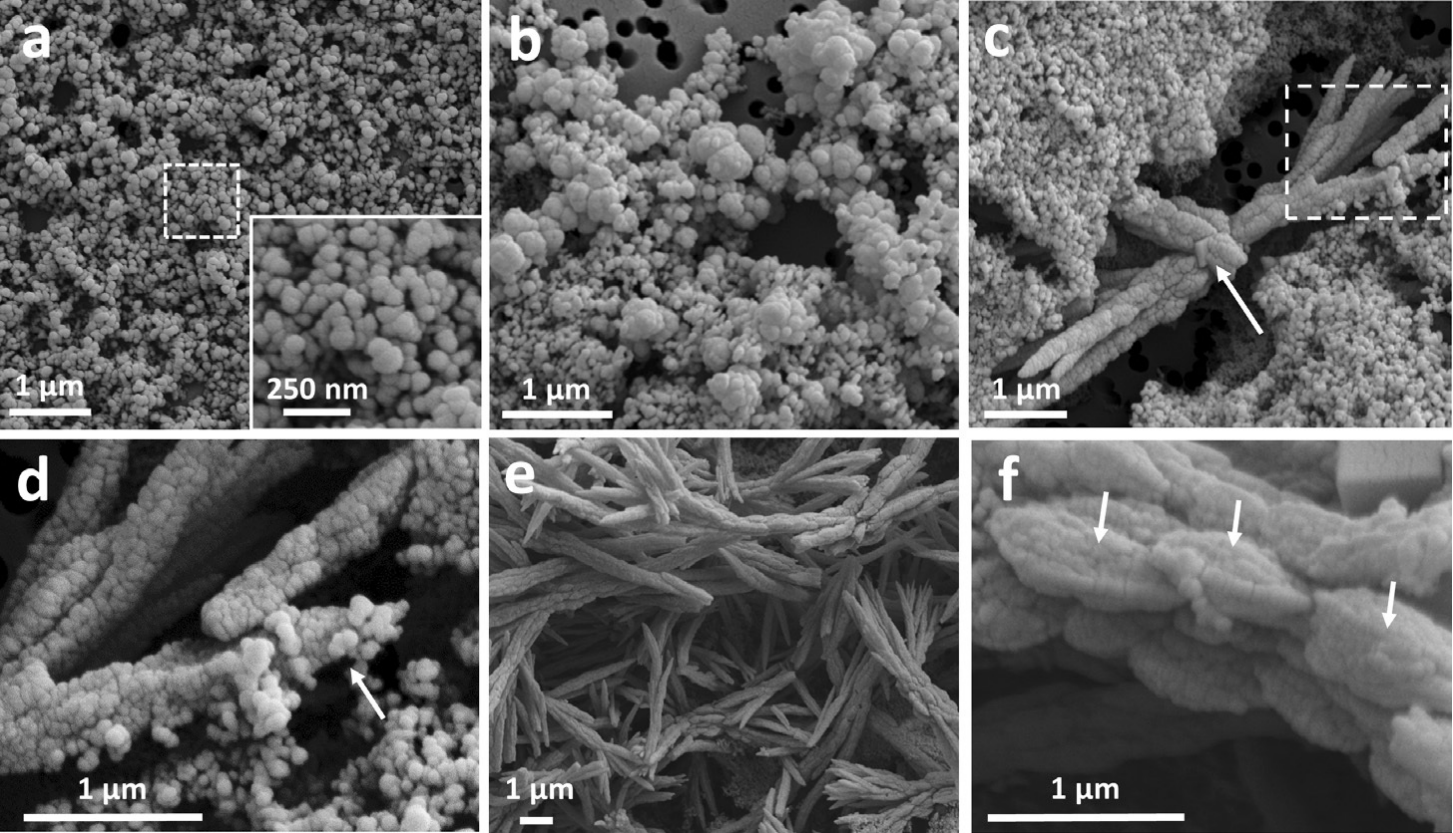
Time‐resolved SEM shows the progression of aragonite crystals, starting from amorphous calcium carbonate (ACC) particles. a) Sample collected after 1 minute of reaction, showing the presence of spherical ACC particles 75–100 nm in size. Inset: higher magnification of the area marked by the dotted square. b) Sample collected after 10 minutes of reaction, depicting 450–600 nm particles. c) Aragonite sheaf‐shaped crystals at 20 minutes of reaction. Arrow: calcite crystal growing at the expense of the surrounding aragonite. Dotted square: area shown in (d) at higher magnification. d) Higher magnification of the area marked by the dotted square in (c), showing spherical ACC particles attaching to the growing aragonite needle (arrow). e) Aragonite needles formed after 1 h of reaction. f) Higher magnification of one aragonite needle formed after 1 h of reaction, showing that they are composed of elongated particles of 900 nm–1 μm (arrows).

In the second stage, after 10 minutes, the reaction contained larger particles, 450–600 nm in size (Figure 2 b), that were further aggregating and arranging into linear structures. The first aragonite “sheaf” crystals were visible after 20 minutes (Figure 2 c), with a continuing presence of small, spherical ACC particles of 60–120 nm in size. The observed topology of the crystal surface was rough and granular (Figure 2 d) which is a characteristic fingerprint of nanoparticle assembly.^11^ Indeed, several small particles can be seen joining the end of a needle (Figure 2 d, arrow). One rhombohedral calcite crystal (Figure 2 c, arrow) was also partially visible, growing at the expense of the surrounding aragonite needles. After 1 hour of reaction, most of the surface was covered by needles of aragonite (Figure 2 e). The polymorph was confirmed by powder X‐ray diffraction and Raman spectroscopy (Supporting Information, Figures S1 and S2). At this stage, the needles were made up of larger, 900 nm–1 μm particles (Figure 2 f, arrows). As aragonite is a kinetic product, it has a fast rate of growth, and large needle or sheaf‐like crystals can form quickly. However, using cryoTEM we have been able to arrest this process, and observe an aragonite needle pair in the early stages of development (white arrows, Figures 3 a). Interestingly, the crystals were composed of nanoparticles (Figure 3 b, white circle), which supports the hypothesis that the needles formed through a particle attachment mechanism (Figure 3 b). This is strongly supported by the time‐resolved SEM measurements, which showed small particles attaching to the needle ends (Figure 2 d).

**Figure 3:**
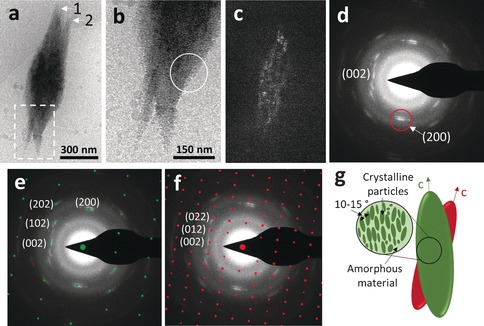
a) CryoTEM image of an aragonite needle pair formed after 1 h of reaction. Arrows indicate two different crystals, superimposed, denoted (1) and (2). Dotted square: area shown in high magnification in (b). b) Higher magnification of the area marked by the dotted square in a), showing a nanoparticulate texture at the edge of the aragonite needle (white circle). c) Dark‐field TEM of a), using the (200) reflection in LDSAED. d) LDSAED of a). Red circle: reflection used for dark field imaging. e) and f) LDSAED pattern overlaid with simulated patterns, showing the presence of two crystals, one oriented perpendicular to the *b*‐axis (e, corresponding to crystal (1)) and one oriented perpendicular to the *a*‐axis (f, corresponding to crystal (2)). g) Representation of the arrangement of the needle pair and co‐existence of amorphous and crystalline material within the crystal. LDSAED patterns are rotated 90° with respect to the bright field image.

LDSAED confirmed that these crystals are composed of aragonite (Figure 3 d). Furthermore, the electron diffraction pattern itself consisted of two overlaid patterns, one for each needle. Simulated patterns shown in Figures 3 e (perpendicular to the *b* axis) and Figures 3 f (perpendicular to the *a* axis) define the reflections that belong to each needle and show that crystal 1 (Figure 3 a) is observed in the *ac* plane whereas crystal 2 is observed in the *bc* plane, with the (002) reflection remaining consistent in both patterns. Furthermore, the reflections in the LDSAED had an angular spread of 10–15°, indicating that both crystals are composed of nanocrystalline domains that are oriented over a 10–15° range.

Dark‐field cryoTEM imaging (Figure 3 c) illuminating the (200) reflection (circle, Figure 3 d) revealed that whilst needle 1 contained nanoscale crystalline material that contributed to the diffraction pattern (denoted by bright regions), these domains were interspersed with dark regions. As these dark regions correspond to areas in the crystals that do not diffract, they must be composed of ACC. Thus, our results show that at this stage, the forming aragonite needles contain crystalline material intermixed with ACC (Figure 3 g). To confirm this, we tested whether crystallization of the amorphous domains could be induced by drying. Indeed, a comparison of the same particle imaged under cryogenic and dry conditions demonstrated that upon freeze‐drying the dark field showed an entirely bright pattern, with no dark regions, confirming that all amorphous domains had crystallized (Supporting Information, Figure S3). Furthermore, the reflections in the diffraction pattern became significantly stronger. Finally, as crystallization was induced by freeze‐drying, the possibility of dissolution of ACC and reprecipitation of aragonite can be excluded, confirming the solid‐state transformation.

As the aragonite needle already has a defined morphology and continues growing through particle attachment, we propose that the interspersed ACC will eventually crystallize into aragonite through a solid‐state transformation. Conventional TEM of a mature aragonite needle, formed after 24 h of reaction, showed a similar LDSAED pattern as in the early stages (Figure 4). However, at this stage the dark field image illuminated with the (002) reflection showed that the whole needle exhibited high contrast, demonstrating that it contains only crystalline material with no detectable ACC. It must be noted that the differences in contrast in the dark‐field images within the individual aragonite needles (Figures 3 c and 4 c) was not related to sample thickness, as both thinner and thicker areas exhibited bright and dark zones (Supporting Information, Figure S4). Whilst previous work has shown ACC acting as source for crystalline phases to grow,^6^ this is the first evidence of both amorphous and crystalline materials co‐existing within an aragonite needle. This combination of phases within the same needle suggests that a solid‐state transformation from amorphous to crystalline is taking place to form a crystal of aragonite. Overall, the data gives a clear view of the progression of mineral growth taking place in this system, an overview of which is shown in Scheme 1. Beginning with small ACC, an aggregation mechanism controls formation of larger particles.

**Figure 4:**
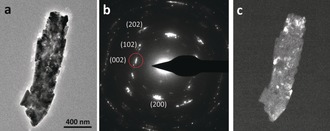
a) Conventional TEM image of a mature aragonite needle after 24 hours of reaction; b) LDSAED pattern of (a). Red circle: reflection used for dark‐field imaging. c) Dark‐field TEM imaging of (a) using the (002) reflection.

**Scheme 1:**
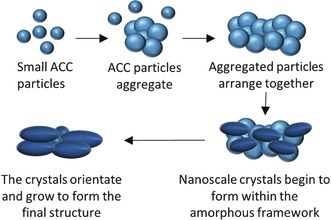
Initial growth mechanism of an aragonite needle in a 50 % ethanol–water mixture. Initially, small ACC particles form, then aggregate into larger particles. These aggregated particles combine to become a larger, linear structure. Nanoscale crystals form within this amorphous framework, which grow to form the final needle structure.

These larger irregular ACC‐clustered particles then aggregate themselves, in a seemingly linear fashion, before crystallization begins through a solid‐state mechanism. This is in contrast to a more classical dissolution–reprecipitation mechanism where ACC dissolves and reprecipitates as aragonite. Additionally, it confirms that the final morphology of aragonite crystals does not provide concrete evidence about their growth mechanism,9e, 10 in agreement with previous work on calcite.^12^ However, it is important to note that more than one mechanism may be present at once in the system, as shown by Nielsen et al.^6^

Biogenic aragonite formation has been shown to form from an ACC precursor, and it has been suggested that it occurs by a non‐classical mechanism,7c, 13 similar to that demonstrated for biogenic calcite.4a, 9d, 14 To date, however, there is no direct confirmation for such pathway in vitro. Here, using a synthetic system, we provide the first evidence for the solid‐state transformation of ACC into aragonite. While our reaction conditions are significantly different from those used by organisms during biomineralization, our results demonstrate that this non‐classical pathway is a possible route for the precipitation of aragonite. The similarity of the particle attachment mechanisms observed in forming biominerals4d, 9e, 15 and in vitro suggests that a similar mechanism is indeed possible in biogenic systems. Further work is required to confirm this hypothesis.

## Conflict of interest

The authors declare no conflict of interest.

## Supporting information

As a service to our authors and readers, this journal provides supporting information supplied by the authors. Such materials are peer reviewed and may be re‐organized for online delivery, but are not copy‐edited or typeset. Technical support issues arising from supporting information (other than missing files) should be addressed to the authors.

Supplementary
