# Supplementary material for: Solid‐State Transformation of Amorphous Calcium Carbonate to Aragonite Captured by CryoTEM
Source: Angew Chem Int Ed Engl. 2017 Aug 15;56(39):11740–3. doi: 10.1002/anie.201703158 (PMC5656811; doi:10.1002/anie.201703158)
Supplement: Supplementary file 1 — Supplementary [file ANIE-56-11740-s001.pdf]

Angewandte Chemie
Eine Zeitschrift der Gesellschaft Deutscher Chemiker

Supporting Information

Solid-State Transformation of Amorphous Calcium Carbonate to Aragonite Captured by CryoTEM

*Jessica M. Walker, Bartosz Marzec, and Fabio Nudelman**

anie_201703158_sm_miscellaneous_information.pdf

Supporting information

Contents

1. **Methods**
   1.1 $CaCO_3$ synthesis
   1.2 Electron microscopy sample preparation
   1.3 pXRD and Raman measurements
2. **Phase determination**
   2.1 P-XRD pattern
   2.2 Raman spectra
3. **Electron Diffraction**
   3.1 Crystallization induced by drying
   3.2 Superposition of darkfield and brightfield images
4. **References**

1 Methods

1.1 Synthesis of $CaCO_3$

For synthesis, stock solutions of $CaCl_2$ and Na_2CO_3 in H_2O (HPLC grade) were prepared. First, Na_2CO_3 was added to the ethanol in a 24 well plate and mixed, before $CaCl_2$ was also added and mixed to form a total reaction volume of 0.5 mL, and a final reaction concentration of 0.025 M of each. An initial white precipitate formed, and the well plate was placed on a rocker for the requisite length of time at 57 oscillations per minute (opm) to replicate the described 'gentle shaking'.[1]

1.2 Electron microscopy sample preparation

For cryoTEM, aliquots (3 µL) of reaction solution were applied to a cryoTEM grid and plunge frozen using a vitrification robot (FEI Vitrobot Mark IV) with the sample application chamber at 21 °C and 100 % humidity. Prior to freezing, cryoTEM grids (Au/C, Quantifoil Micro Tools GmbH) with 2 µm holes were plasma treated using a Quorumtech Glow Discharge system for 45 s.

For conventional TEM, the reaction solution was filtered after 24 hours using a 0.22 µm membrane (Millipore) and washed with ethanol. The filtrate was resuspended in ethanol before 3 µL was applied to a 200 mesh C/Ni grid and left to dry.

A FEI F20 Technai electron microscope with 200 keV field emission gun, equipped with a Gatan cryoholder operating at ca. -170 °C was used for imaging and LDSAED. Images were recorded on an 8k x 8k CMOS TVIPS F816 camera.

For SEM, aliquots (10 µL) of reaction solution were removed at various timepoints up to 60 minutes, filtered under vacuum through a 0.22 µm filter membrane (Millipore) and washed with ethanol. Samples were sputter-coated with carbon before being imaged using a Zeiss Sigma HD VP Field emission scanning electron microscope.

1.3 PXRD and Raman measurements

Powder XRD was taken from crystals obtained by filtering the whole reaction solution and measuring using a 0.5 mm wall glass capillary on a Bruker D8-Advance X-ray Diffractometer with a Cu Kα lamp (λ=1.54056 Å).

Crystals precipitated from solution on to a glass slide were used to obtain Raman spectra, measured using a Renishaw InVia Raman microscope with a wavelength of 785 nm.

2 Phase determination

2.1 Powder X-ray diffraction

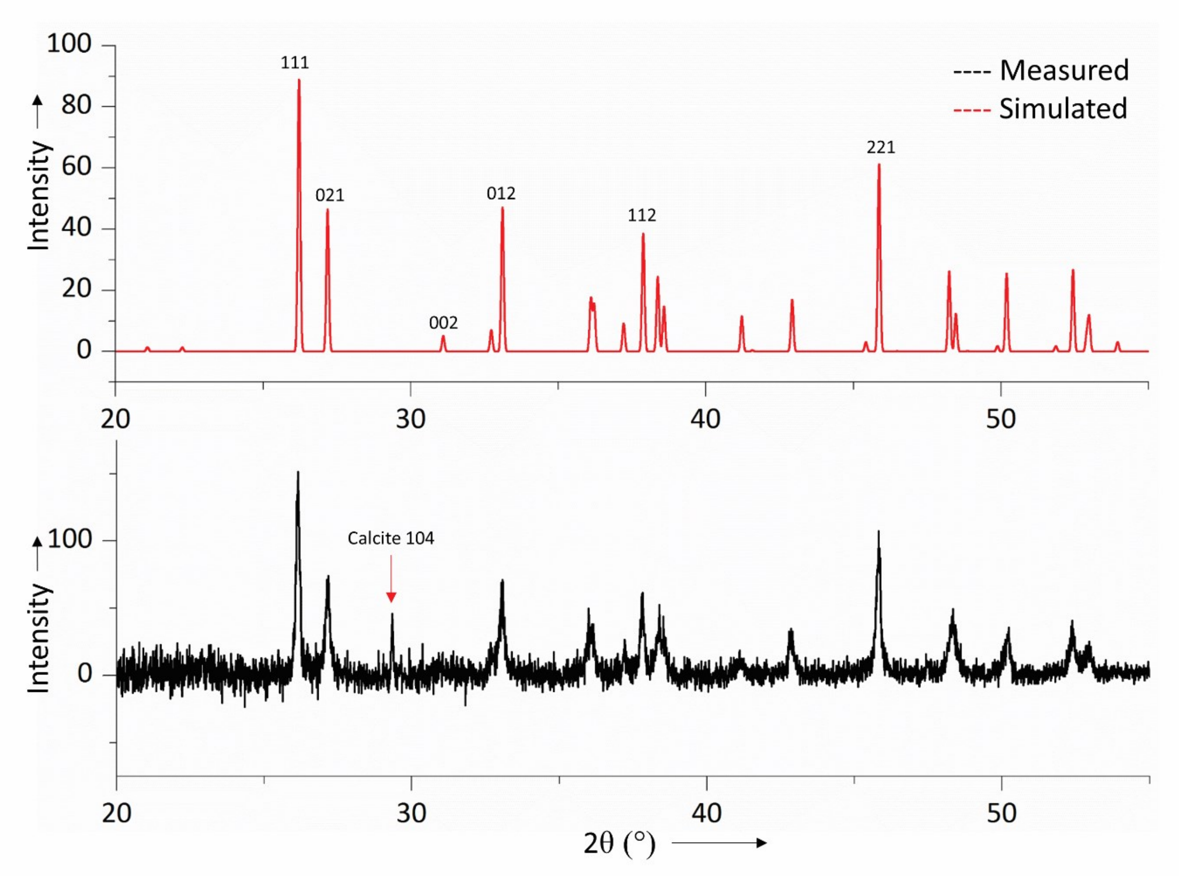

Figure S1 An aragonite XRD pattern simulated from unit cell data[2] with peaks annotated with associated planes (red, top). The measured powder XRD pattern (bottom, black) was taken from the whole reaction filtered on to a 0.22 µm filter membrane and transferred to a capillary. It shows aragonite and a small amount of calcite (104 plane at 29.1 °). As the 104 plane of calcite diffracts strongly, this peak accounts for the presence of only a small amount of calcite.

2.2 Raman Spectroscopy

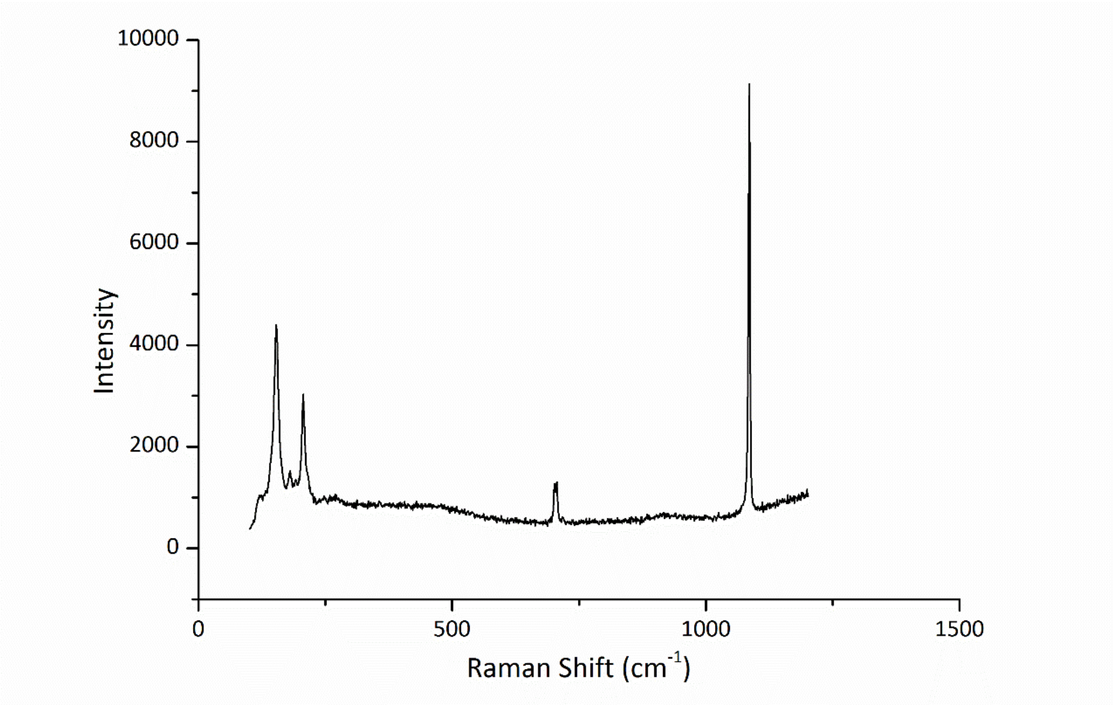

Figure S2. Raman pattern from an aragonite crystal precipitated onto a glass slide

3 Electron diffraction

3.2 Crystallization induced by drying

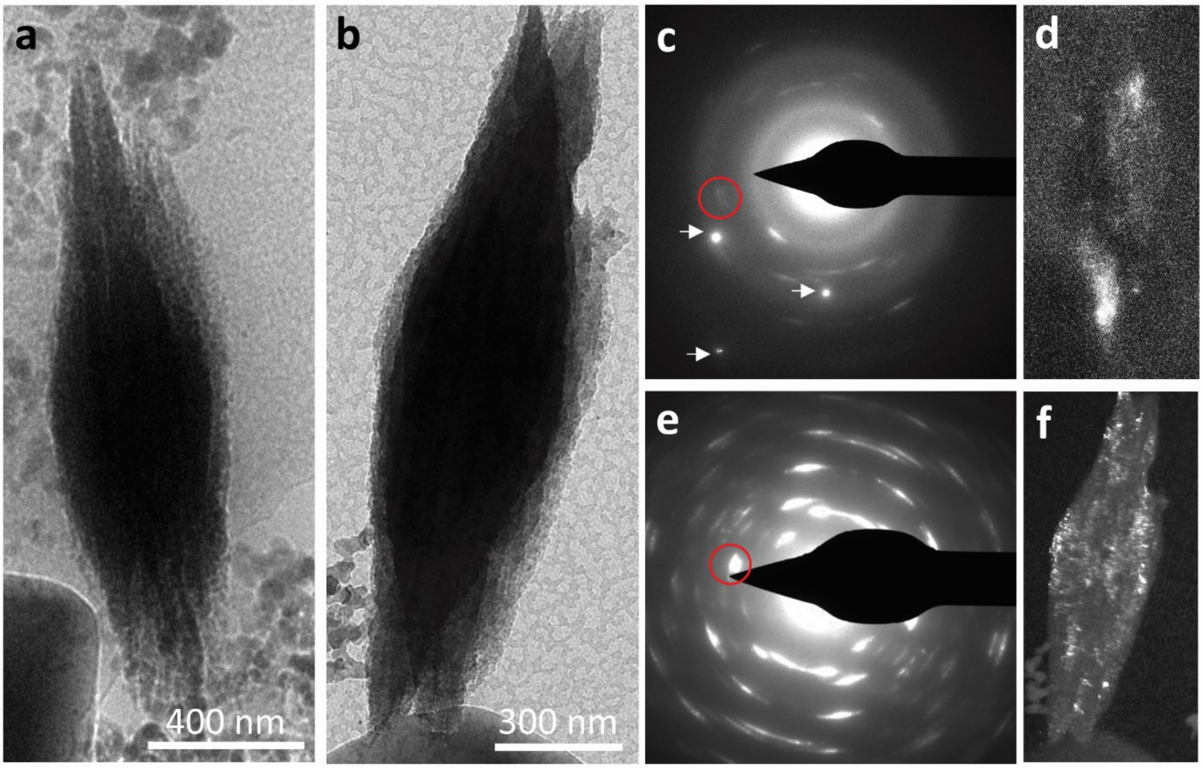

Figure S3. a) A frozen aragonite needle after 1 hour of reaction (ice crystal identified by white arrow). b) The same needle as a) shown after drying to induce crystallization. c) The electron diffraction pattern of a) consistent with that of aragonite. Highlighted in red is the spot responsible for the darkfield image (d) corresponding to the *(200)*-reflection. Bright spots highlighted by white arrows correspond to the ice crystal shown in a). d) Darkfield image of the diffraction spot marked in c) showing interspersed dark and light regions suggesting a mixture of crystalline and non-crystalline material. e) LDSAED of b) showing a similar aragonite diffraction pattern to c) and highlighting the *(200)*-reflection used for f). f) Dark field image taken from e) shows a now fully bright crystal. This shows that crystallization has taken place in the dark regions induced by drying to make a fully crystalline needle.

Brightfield and darkfield images were rotated 90° for clarity.

3.3 Superposition of darkfield and brightfield images from Figure 3 and 4

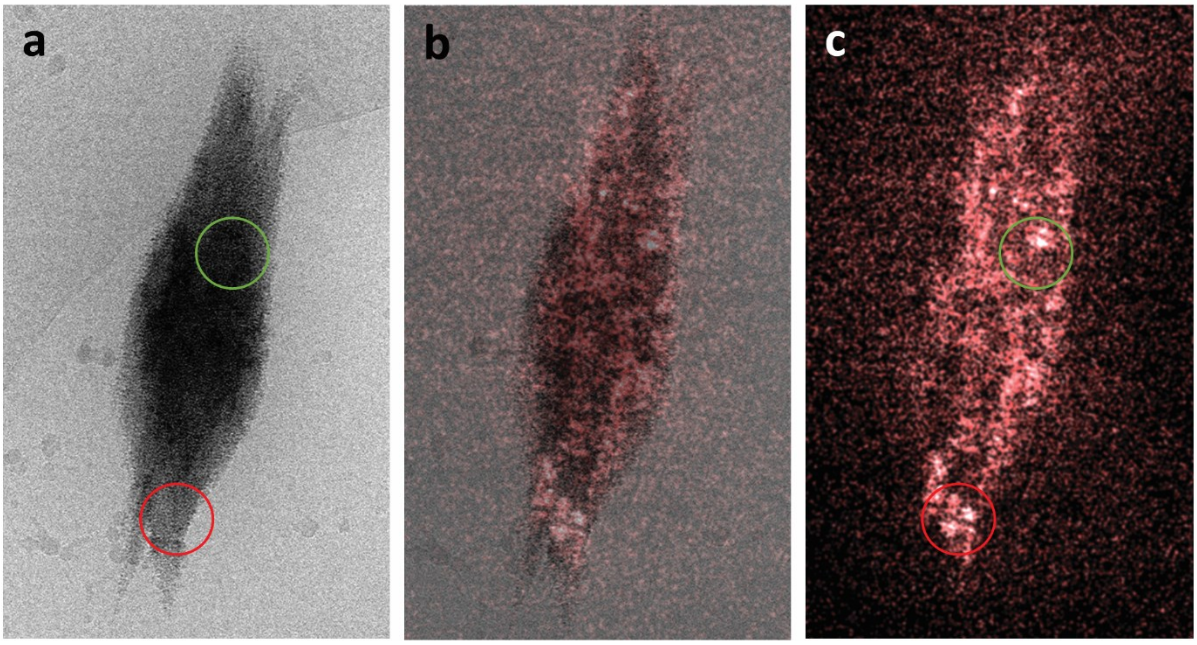

Figure S4. a) Brightfield image of particle viewed after 60 minutes in cryoTEM with a low contrast area highlighted by a red circle and a high contrast area highlighted by a green circle. b) Superposition of the dark and brightfield images of the particle. c) Darkfield image of the same crystal with corresponding areas to a) highlighted, showing that bright areas can be related to both low and high contrast regions.

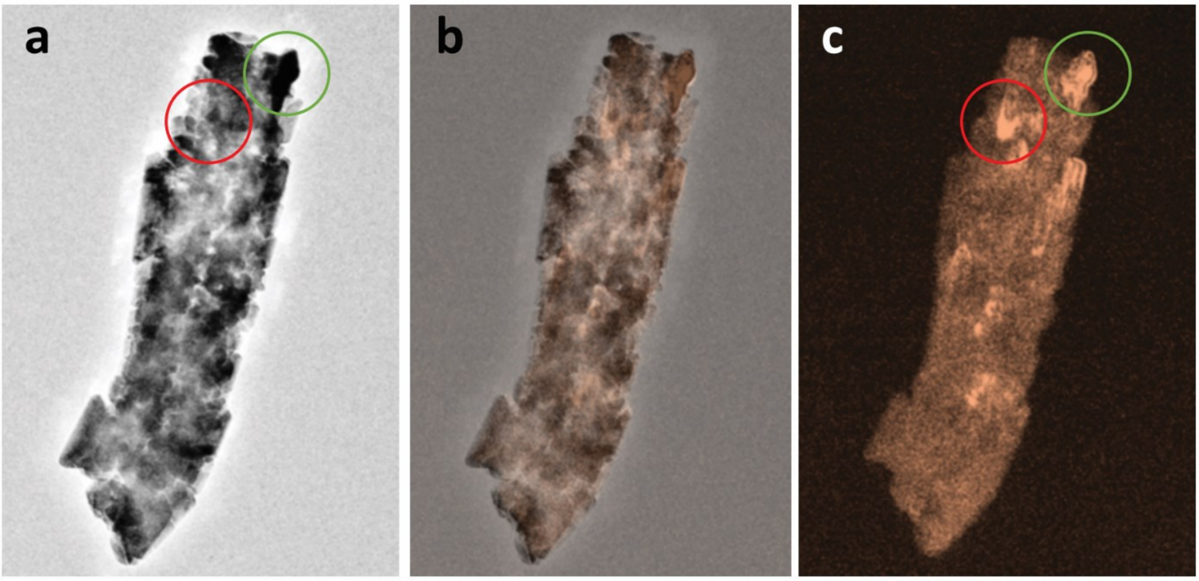

Figure S5. a) Brightfield image of an aragonite crystal after 24 hours in conventional TEM with an area of less contrast highlighted by a red circle and an area of higher contrast highlighted by a green circle. b) Superposition of the dark and brightfield images. c) Darkfield image of the same crystals with the corresponding areas to a) highlighted, showing both contain similarly bright patches despite the change in contrast.

4 References

[1] K. K. Sand, J. D. Rodriguez, E. Makovicky, L. G. Benning, S. L. S. Stipp, *Cryst. Growth Des.* **2012**, *12*, 842-853.

[2] S. M. Antao, I. Hassan, *The Canadian Mineralogist* **2010**, *48*, 1225-1236.
